# Mask Responses for Single-Pixel Terahertz Imaging

**DOI:** 10.1038/s41598-018-23313-6

**Published:** 2018-03-20

**Authors:** Sven Augustin, Sven Frohmann, Peter Jung, Heinz-Wilhelm Hübers

**Affiliations:** 1 Department of Physics, Humboldt Universität zu Berlin, Berlin, 12489 Germany; 2 Department of Optical Sensor Systems, German Aerospace Center Berlin - Adlershof, Berlin, 12489 Germany; 3 Communications and Information Theory Group, Technical University Berlin, Berlin, 10587 Germany

## Abstract

Terahertz (THz) radiation meaning electromagnetic radiation in the range from 0.1 (3) to 10 (30) has the unique advantage of easily penetrating many obstructions while being non-hazardous to organic tissue since it is non-ionizing. A shortcoming of this domain is the limited availability of high-sensitivity detector arrays respective THz cameras with >1k pixels. To overcome the imaging limitations of the THz domain, compressive imaging in combination with an optically controllable THz spatial light modulator is a promising approach especially when used in a single-pixel imaging modality. The imaging fidelity, performance and speed of this approach depend crucially on the imaging patterns also called masks and their properties used in the imaging process. Therefore, in this paper, it is investigated how the image quality after reconstruction is specifically influenced by the different mask types and their properties in a compressive imaging modality. The evaluation uses an liquid-crystal display based projector as spatial light modulator to derive specific guidelines for the use of binary and true greyscale masks in THz single-pixel imaging setups respective THz single-pixel cameras when used in far-field applications e.g. stand-off security imaging.

## Introduction

Imaging at low terahertz (THz) frequencies provides unique information in particular for applications in security imaging because at these frequencies a good compromise between penetration properties on one hand and spatial resolution on the other hand is possible. As a rule of thumb, the penetration properties greatly improve with decreasing THz frequency. However, the spatial resolution decreases at the same time. Another challenge is the lack of suitable multi-pixel THz cameras. Accordingly, the classical approach for security imaging at frequencies in the low THz region (below 1) involves some form of mechanical scanning either with a single or a few detector elements^1,2^. Due to the mechanical scanning the achievable frame rate is rather limited depending on the amount of necessary image pixels. To overcome the frame rate limitation the combination of a sensitive single-pixel detector with compressive imaging offers a potential solution.

Compressive imaging refers to an imaging process in which images can be acquired with fewer measurements than image pixels. This can either be intentional (compression) or unintentional (e.g. measurement errors). We focus here on the unintentional case where in a far-field single-pixel imaging system random measurement errors occur. Therefore, different mask types are investigated that allow for robust image reconstruction in this particular compressive imaging case.

One way to implement such a measurement scheme is the use of a spatial light modulator (SLM). When a SLM is combined with a single-pixel detector such an imaging setup is usually referred to as a single-pixel camera (SPC). The SPC concept (in this sense) for the visible part of the electromagnetic spectrum (VIS-SPC) was introduced in 2008^3^. The general design of a SPC enables image acquisition with increased resolution^4^, improved signal-to-noise ratio (SNR)^5^ and larger depth of focus^6^. All these advantages can be achieved with a single-pixel detector even without mechanical scanning with the help of a SLM, which, in this case, acts as a dynamic aperture^7^. An image of the scene is formed from sequential measurements and an image reconstruction process that is based on solving an (underdetermined) system of equations (image reconstruction)^8^). The SPC concept is especially beneficial for cameras that work outside of the visible part of the electromagnetic spectrum (EM-spectrum) respective, for example for the THz domain.

**Figure 1:**
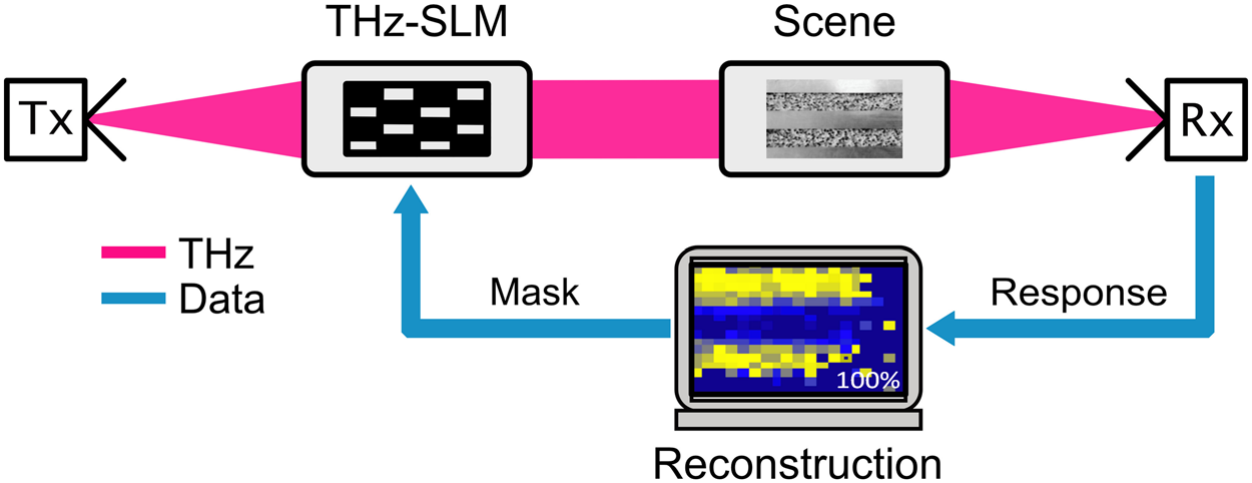
Scheme of the imaging process encountered in a THz-SPC. The beam coming from the THz source (Tx) is spatially modulated by a THz-SLM. The spatially modulated THz beam is directed to the scene of the camera and the radiation coming from the scene is detected using a single-pixel detector (Rx). With the knowledge of the spatial modulation patterns in connection with the measured responses an image of the scene can be reconstructed using a non-linear reconstruction algorithm.

Following the VIS-SPC concept, similar implementations in the THz domain have been realized and implementations for this domain are reported in^9–13^. In these systems a THz-time-domain system^9,13^, a 0.35 THz multiplier source^11^ or an incandescent light source^10^ was used as a source of THz radiation. The general design of a SPC is similar for the VIS and the THz domain. It consists of a SLM, a radiation source and a single-pixel detector. A major difference between a VIS-SPC and a THz-SPC is the implementation of its SLM since THz-SLMs are not readily available. There are for example, THz-SLMs that are still in the early research stage, which are based on metamaterials as described in^12^. This THz-SLM approach addresses individual pixels electronically. While it makes such SLMs easy to control this approach has the shortcoming of providing only a very small amount of controllable pixels (so far _<_100 pixels were reported). Another approach that allows for more than 1500 individually controllable pixels is an optically controllable THz-SLM. In this approach a VIS-SLM is used to project spatial patterns (so called masks) onto a suitable semiconductor disc, the optical switch (OS). In the illuminated regions electrons are excited into the conduction band and the semiconductor (partially) changes from a semiconducting to a metallic state becoming less transmissive for THz radiation. This mechanism implies that the performance of an optically controllable THz-SLM is influenced by the quality and quantity of the OS’™s illumination and the OS material (the type and quality of the semiconductor) itself^14,15^. A detailed analysis of this subject, while extremely relevant for THz-SPCs, is beyond the scope of the analysis presented here. Figure 1 shows a generic block diagram of an optically controllable THz-SPC visualizing the concept just presented.

In this paper we investigate relevant mask properties used for 0.35-SPC imaging in an optical modulation approach. This approach is known in the scientific community for some time^16^ and was recently used for THz near-field imaging quite successfully^17,18^. Since the mask properties are of such importance for the image quality and all published work in this area has so far not focused on different mask types in an unintentional compressive case (as introduced above). Accordingly in our investigation, Gaussian, Bernoulli, Hadamard and Discrete Cosine Transform masks are investigated in a 0.35 far-field single-pixel imaging system regarding robustness against measurement error. The next section presents the reconstruction results for the different mask type measurements. As first result a single metallic edge is evaluated when fewer randomly chosen measurements than image pixels are acquired (compressive imaging modality). The measurements are chosen at random since this case is closer to the situation that is encountered in stand-off imaging. This first step of the analysis will identify pseudo-random Bernoulli masks as a good choice for the envisioned imaging scenario. As evidence this mask type is used in the second step of the analysis for the compressive imaging of a metallic test target that is several centimeters large and imaged also several centimeters from the THz-SLM (far-field). After the presentation of the results they are discussed and conclusions are drawn. The last section entitled “Methods” then presents the details of the experimental method and procedure to enable the reader to reproduce the results presented in this text.

## Results

The reconstruction algorithm is used to solve the linearized imaging model stated in Equation (1). As known, the algorithm that is used to calculate an image from the measured THz responses has significant influence on the reconstruction performance in terms of image fidelity, reconstruction speed, achievable compression factor, etc.

1 $$Y={\rm{\Phi }}\cdot X+N$$

Here, Y denotes the vector of measured mask responses and X is the THz representation of the scene to be reconstructed. The matrix Φ contains in each row the mask used for the respective measurement *Y*_*i*_ and the vector N models an additive Gaussian noise contribution. For the evaluation of the results Equation (1) is solved using a least-square algorithm with the additional constraint of non-negativity of the measured values *Y*_*i*_ (Non-Negative Least Squares NNLS). NNLS was chosen due to its robustness properties in a compressive imaging modality. The performance of the NNLS algorithm was analyzed for binary Bernoulli masks here^19,20^. The performance of NNLS for Hadamard and greyscale masks is still an open research topic^21^.

**Figure 2:**
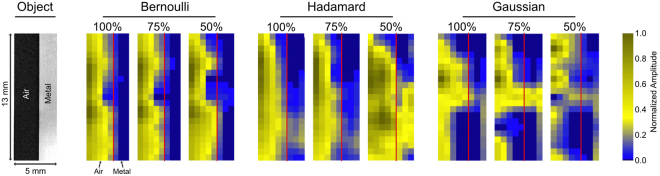
Reconstructions of a metallic edge using the NNLS algorithm for Bernoulli masks, Hadamard masks and Gaussian masks. A reconstruction using DCT masks was unsuccessful and is therefore not shown here. In each case the 100% case uses a number of measurements that is equal to the number of pixels. The size of the metallic edge is 13 in height and 5 in width (see photo on the left hand side for details).

**Figure 3:**
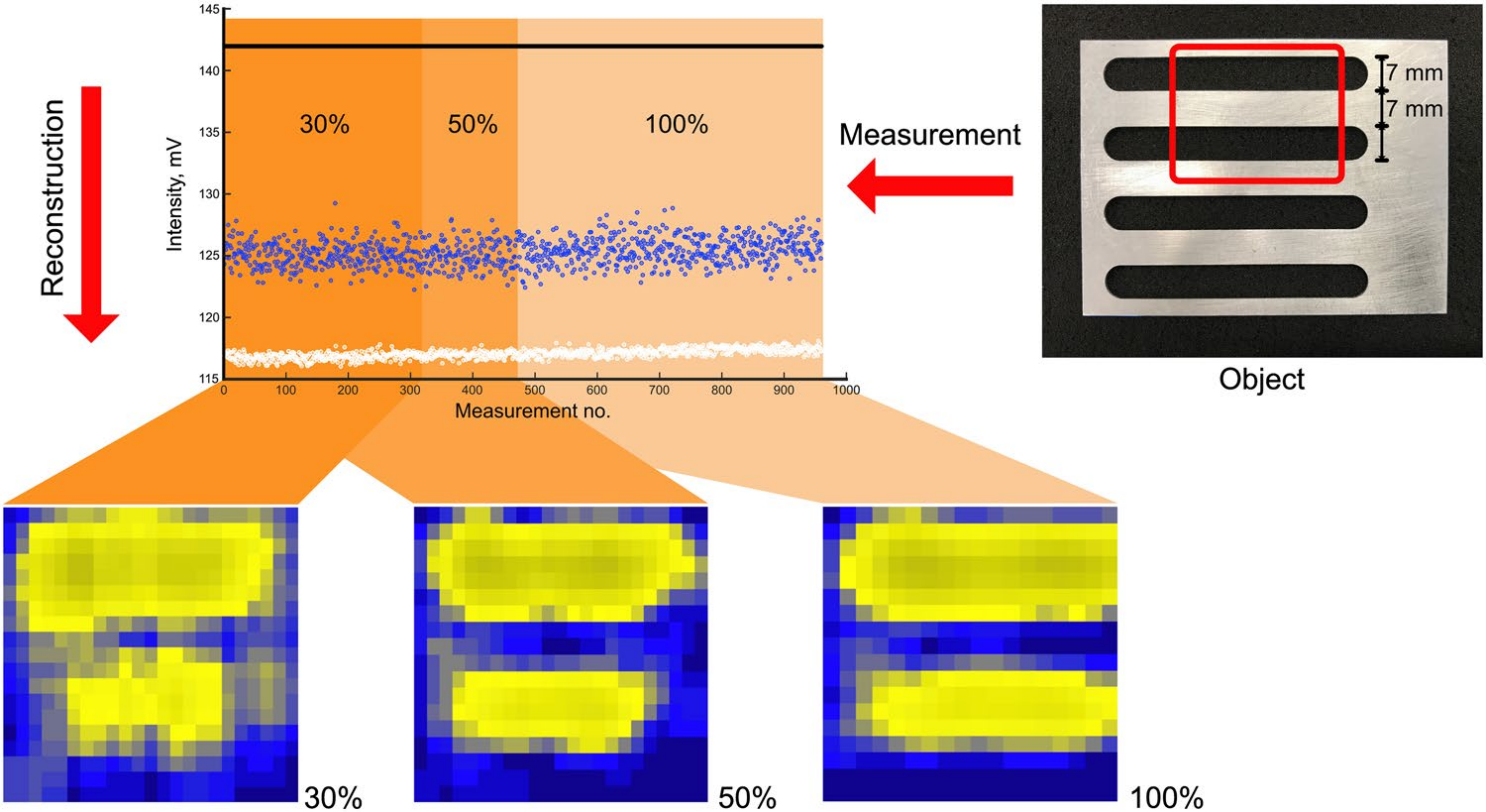
Imaged metal grid scene target using Bernoulli masks. Shown are the image reconstructions with a reduced number of considered measurements (100–30% when Bernoulli masks are commanded to the SPC. The scene target was a metallic grid. The target has 7 wide metallic bars and 7 mm wide open spaces (binary target - see photo for details).

Figure 2 shows the results of the mask type comparison. Only Bernoulli and Hadamard masks are able to produce a reasonably good image of the single metallic edge shown in the left-hand side of Fig. 2. The Gaussian masks produce a THz representation of the edge that shows already significant distortions in the case where the number of measurements is equal to the number of pixels (100%). In the compressive cases (number of measurements <100% randomly chosen values *Y*_*i*_ are omitted for the reconstruction process (a detailed description of the signal processing method that was used to simulate the compressive imaging modality can be found in the Methods section). As can also be seen in Fig. 2 only the Bernoulli masks show good compression results of the metallic edge even when only random 50% of the measured values are used for reconstruction. According to this analysis, only the pseudo-random Bernoulli masks exhibit potential for compressive imaging. As evidence for this conclusion a metallic grid object imaged with the 0.35 THz SPC in a compressive modality is shown in Fig. 3.

Even with only 30% of all masks the shape of the imaged object is recognizable albeit with smaller SNR. The hypothesis that Bernoulli masks are well-suited for a compressive imaging modality is clearly supported by the result shown in Fig. 3. Although the SNR is smaller for the 30% compression case and the metal edges are smeared out the structure is still recognizable. The result might even be improved using a sparsifying transformation^22^. Such a transformation could not be applied to the results presented here since sparsifying transformations for 0.35 are still an open research subject.

## Discussion

To summarize and discuss the results of the investigation just presented, specific guidelines for SPC measurements can be derived. The Hadamard mask measurements provide a large modulation for specific masks but in a compressive imaging modality, where a random part of the measurements is not considered for reconstruction, their application was found to be not optimal. The compressive modality (fewer measurements than image pixels due to measurement errors) is often encountered in far-field 0.35 single-pixel imaging applications. The measurement errors can arise due to output power drifts of the Tx itself, the coherent nature of the radiation causing interference effects or even standing waves. This is especially true in cases where the scene is several centimeters or even meters away from the optical switch (security imaging applications).

Mask measurements acquired under the aforementioned conditions lead to measurement errors (transmission errors) that should be omitted for the reconstruction. This approach will automatically lead to a compressive imaging modality. Due to this reason the use of pseudo-random masks is supported here. The performance of pseudo-random masks under compressive conditions can even be improved with a SNR like quantity, which would provide an indicator whether a SPC measurement was successful even without image reconstruction. This quantity should even give a quantitative measure for the decision which mask measurements can be considered for reconstruction.

As shown by the investigation presented here only pseudo-random mask type measurements give sufficient compressibility in the pixel domain. The compressibility of pseudo-random masks coincides with a robustness property against transmission/measurement error^23^. It is assumed that this property stems from the ability of pseudo-random masks to acquire information content of the entire scene with each measurement. This means that the image content is not diminished with increasing undersampling factors only the SNR of the reconstructed images. Compared with a traditional (software) raster scan the increase in SNR and robustness of pseudo-random mask measurements theoretically outweigh the raster scan approach by several orders of magnitude. The exact figure depends on the number of image pixels, details of the SPC implementation as well as the information content of the scene target. As a rule of thumb, the SNR increase is of the order N/2 where N is the number of image pixels.

However, the question “what are the best masks?” can only be answered knowing the specific imaging task at hand. Additionally, the identified beneficial properties of pseudo-random masks can be significantly improved with the help of sparse domains, in other words, transformations of the scene that significantly improve its sparsity. For 0.35 single-pixel imaging such domains are still an open research topic. Shearlets might be an adequate way to find a solution for this task^24^ due to the cartoon-like nature of images in this part of the EM-spectrum.

The analysis also suggests that the use of greyscale masks is not possible for 0.35 THz-SPCs without a suitable calibration of the camera. The calibration would also be beneficial in terms of reducing measurement overhead and towards imaging greyscale targets. First approaches on the subject are mentioned here^25^ and may prove helpful. As long as no calibration procedure for 0.35 THz-SPCs exists, imaging is confined to binary masks and binary objects. As stated before, the robustness and compressibility properties of pseudo-random masks (Bernoulli masks) are very beneficial for the imaging process. Still, the reconstruction approaches are still lacking reconstruction speed. When real-time reconstruction speed is necessary Hadamard masks are still a good choice.

As a brief outlook, so far the investigation excluded the dimension of mask block size. A larger block size increases measurement SNR but on the other hand decreases the achievable spatial resolution in the reconstructed images. Additionally, the effect due to the coherent nature of the THz radiation was not considered and probably plays a role in relation to the mask block size. Since this may be a limiting factor for image fidelity and image resolution, it will be considered in future experiments. The goal of these future experiments is the successful application of the THz-SPC concept for real world (greyscale) targets. For this application a reflection modality even allows security imaging applications, which is especially useful when combined with a radar approach^26^.

## Methods

### Experimental Setup and Procedure

**Figure 4:**
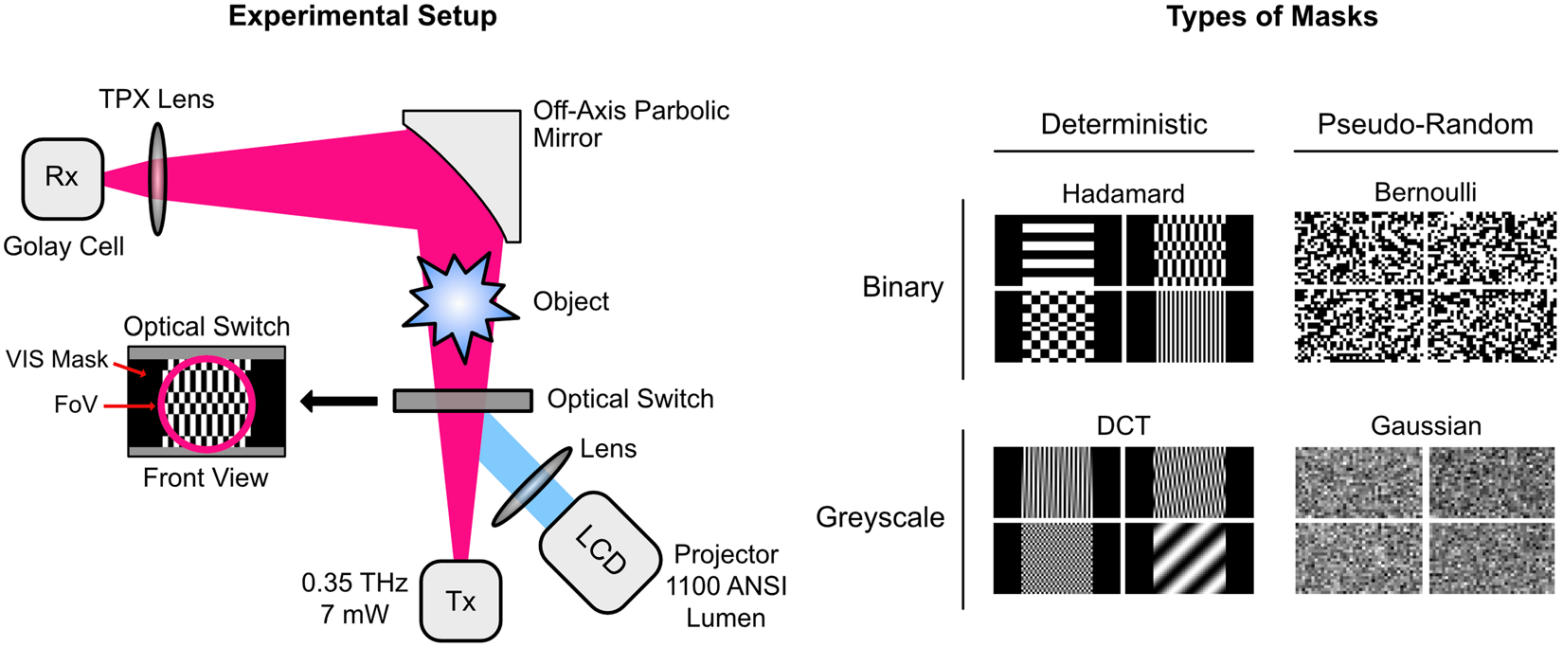
Single-pixel camera setup used for the measurements presented in the text (left-hand side). The THz-SPC in this case uses a commercially available projector as VIS-SLM and light source. The right-hand side shows the categorization with example masks investigated here.

The experimental setup is shown in Fig. 4. The transmitter (Tx) is based on a Yttrium Iron Garnet oscillator, which is amplified and multiplied to 7 of output power at a frequency of 0.35. A horn antenna at the output of the Tx delivers a Gaussian-shaped beam, which impinges directly onto the OS made from passivated silicon (thickness 150 provided by Solarworld AG). This type of silicon is especially sensitive to visible light (VIS), which enables the use of a commercial liquid crystal based projector as VIS light source. This projector enables the display of true greyscale masks due to its liquid crystal display SLM. To enable efficient coupling of the THz and the VIS radiation both optical paths are aligned at an angle of 45 with respect to each other. This enables a very compact design and increases the modulation but introduces geometrical distortions into the projected masks. An alternative design would be the use of a dichroic mirror e.g. made from glass coated with indium tin oxide. Such a mirror was tested but its use caused a reduction of modulation of the OS by a few percent. Therefore, the 45 geometry was used throughout the experiments. The VIS light intensity was estimated to be 1100 ANSI lumen −30% loss introduced by the various optical elements and slightly larger illumination of the OS than the size of the THz beam. The THz radiation that passes through the OS is collected using a custom made off-axis parabolic mirror in combination with a lens made from Polymethylpentene (TPX). This collecting optics was designed in such a way that the entire THz radiation passing through the OS is focused onto a single-pixel Golay cell detector.

The field-of-view (FoV) of this single-pixel camera was approximately 9.5 cm^2^. It is mainly limited by the beam shape of the THz beam at the OS and not by the size of the OS itself. A projector was used to project spatial patterns (masks) onto the OS. In the illuminated regions the semiconductor (partially) changes from a semiconducting to a metallic state and becomes less transmissive for the 0.35 radiation impinging on the illuminated OS. The degree by which the THz transmission is changed upon illumination with a mask, i.e. the maximum modulation M, is evaluated using Equation (2).

2 $$M=1-\frac{{I}_{white}}{{I}_{black}}$$

The maximum modulation (M) results from commanding an entirely white respective black mask to the THz-SPC. The resulting intensities are named *I*_*white*_ and *I*_*black*_. In the absence of interference effects the measured intensity of spatially structured masks lies in between. With the setup described above, single-pixel imaging capability for 0.35 radiation is achieved with a maximum modulation M of approx. 15%.

### Mask Types and THz Responses

As already mentioned, the quality and type of different spatially structured masks directly influences the imaging performance in terms of achievable resolution, fidelity, imaging speed, compressibility, etc. Due to the implementation approach of the THz-SPC using a liquid crystal based projector, a vast selection of masks, binary or greyscale, can be used. To establish a systematic approach, the masks are classified here into four major categories. The first distinction is in regard to the mask type describing the fundamental structure. Here, two categories are used; the deterministic mask type and the pseudo-random mask type. All possible masks cannot be classified this way; since also masks exist that are a mixture of the deterministic and pseudo-random category. For both categories the masks can additionally be categorized as either binary or greyscale. In binary masks each pixel can be either fully transmissive (1) or not-transmissive (0), while in greyscale masks the transmission of each pixel can vary between 0 and 1. These four categories are investigated here using a prominent example for each category. This categorization including each investigated example is shown on the right-hand side of Fig. 4. In the following, the main features of these masks are described. Note that the experiment was designed in such a way that the minimum physical block size in the masks was chosen to approx. 4 × λ ≈ 4 to minimise diffraction effects.

### Hadamard masks

They exhibit a periodic structure that increases in spatial frequency with increasing mask number (mask order used for the experiments here). For the generation of the Hadamard masks used for the investigation presented here, the built-in functions of the MATLAB programming language were used. All masks are rescaled to the interval [0, 255]. The periodic structure of the Hadamard masks gives a large correlation in the imaging process only with a limited number of spatial frequencies in the scene.

### Bernoulli masks

Due to the pseudo-random structure of these masks each measurement gives information about large spatial frequency content of the scene. Thereby the Bernoulli masks are generated by choosing at random a value of 1 or 0 for each mask block. Each mask block is comprised of multiple SLM pixels. Again for the physical implementation the masks are rescaled to the interval [0, 255].

### DCT masks

The abbreviation DCT stands for Discrete Cosine Transform. These masks are greyscale, deterministic masks that contain multiple values in the interval [−1, 1]. The DCT masks as deterministic, greyscale mask type example investigated here, are also generated with the help of built-in MATLAB functions and are rescaled to the interval [0, 255]. The performance of DCT masks is especially interesting since the results can be related to the JPEG compression standard.

### Gaussian masks

The last mask type investigated here uses Gaussian masks, which are masks where each mask block is drawn separately from a standard Gaussian distribution. The result is then rescaled to the interval [0, 255].

**Figure 5:**
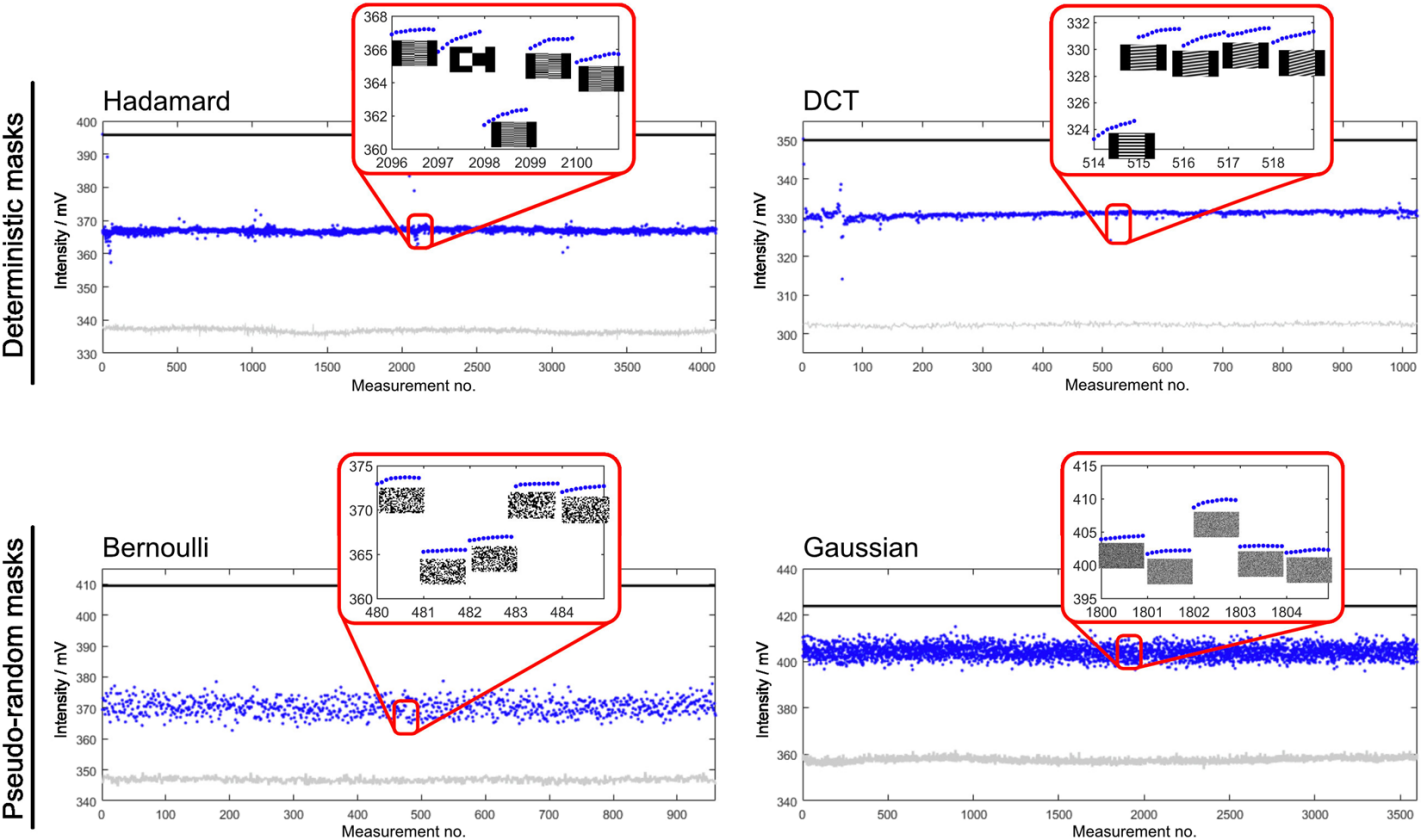
Mask responses of the different mask types investigated here. In each case a representative subset illustrating the specific responses is shown. The inset in the diagram shows various example masks and that for each measured mask ten individual values are acquired.

Both greyscale mask type examples are of special theoretical interest since they allow the derivation of theoretical imaging limits (Gaussian masks) and come with fast reconstruction algorithms (DCT - non-compressive). Due to the fundamentally different nature of the four mask types, different THz-responses are expected when they are commanded to the THz-SPC. Each response is also influenced by the number of mask pixels with the same value (mask block size) and whether the structured part of the masks has to be quadratic or rectangular (orthogonal transforms). The responses, i.e. the intensity measured with the Golay detector for each commanded mask, of the four mask types are shown in Fig. 5 as a function of the measurement number.

Here, each measurement number corresponds to one particular mask of the respective category of masks. In each of the plots the upper black curve corresponds to the signal measured with the Golay detector when a completely black mask is commanded to the projector (i.e. no illumination of the semiconductor and maximum transmission of the THz beam) and the lower gray curve corresponds to the THz signal when a completely white mask is projected (i.e. the whole semiconductor is illuminated by the projector). The blue curve is the signal measured with the Golay detector when the semiconductor is illuminated with a spatially structured mask. In the insets some examples of commanded spatially structured masks and the corresponding THz signals are shown.

There are a number of general features which can be seen in Fig. 5. First of all the intensity measured with the Hadamard and DCT masks strongly deviate from the average value only for a few specific masks. This corresponds to the fact that these masks have a strong correlation with the scene. The THz responses for the pseudo-random masks have a significantly larger average variation than those of the deterministic masks. However, unlike the Hadamard and DCT masks no value stands out. This fundamental difference in the measured responses indicates that the sequence of masks in a SPC imaging process is only important for deterministic masks and that the pseudo-random masks capture, with each measurement, large information content of the scene. It can also be seen that the average intensity is different for the various example mask types. This is likely due to the changing coupling efficiency caused by interference effects in the OS (beam steering).

In order to simulate the compressive imaging modality for all image acquisitions with the THz-SPC, the number of measured structured masks was equal to the number of pixels. From these measurements a portion commensurate to the undersampling ratio/compression ratio was selected at random. Only these measurements were considered in the reconstruction process. Additionally, for each structured mask two unstructured masks (black, white) were measured in order to account for power drifts during the measurements. This drift correction was implemented using the assumption that the value for the measured black masks is constant throughout the entire THz-SPC imaging process. This assumption gives a scaling factor for each structured mask.
